# Mental health challenges and perceptions of stigma among youth living with HIV in Tanzania

**DOI:** 10.1371/journal.pone.0318035

**Published:** 2025-01-28

**Authors:** Laura S. Mkumba, Fortunata Nasuwa, Blandina T. Mmbaga, Aisa M. Shayo, Coleen K. Cunningham, Karen E. O’Donnell, Dorothy E. Dow

**Affiliations:** 1 Duke Global Health Institute, Durham, North Carolina, United States of America; 2 Kilimanjaro Christian Medical Center, Moshi, Tanzania; 3 Kilimanjaro Christian Medical University College, Moshi, Tanzania; 4 Department of Pediatrics, Division of Infectious Diseases, Duke University Medical Center, Durham, North Carolina United States of America; 5 Department of Pediatrics, University of California, Irvine, Orange, CA, United States of America; 6 Children’s Hospital of Orange County, Orange, CA, United States of America; 7 Center for Health Policy and Inequalities Research, Duke Global Health Institute, Durham, North Carolina, United States of America; 8 Center for Child and Family Health, Duke University, Durham, North Carolina, United States of America; Emory University School of Medicine, UNITED STATES OF AMERICA

## Abstract

Youth living with HIV (YLWH) face psychosocial challenges and HIV-related stigma, which impact adherence to antiretroviral therapy (ART). This study was designed to understand better the change in mental health symptoms and experiences with stigma among YLWH in Tanzania who completed the original pilot *Sauti ya Vijana* (*SYV*), a mental health and life skills group intervention. YLWH who completed *SYV* and demonstrated a change of ≥2 points in either direction on their Patient Health Questionnaire PHQ-9 (depression screener) from baseline to 18 months were purposively sampled. HIV Stigma was measured using 10-questions from the Berger HIV Stigma Scale, and findings ranged from 4–16 for internal stigma and 6–24 for external stigma. In-depth interviews (IDI) were conducted in Kiswahili and included topics such as history of mental health challenges, perceptions of stigma, and experiences with the *SYV* intervention. Interviews were transcribed, translated to English, and analyzed for emergent themes. Ten youth, 18–25 years of age, were interviewed; 70% were male. Mean (SD) PHQ-9 scores were 7.3 (SD = 3.5) at baseline and 5.6 (SD = 5.0) at 18 months. All participants reported experiencing intermittent episodes of mental health challenges due to difficult interpersonal relationships and fear of stigma. Youth relied on peer support and skills from the *SYV* intervention to cope with mental health challenges and stigma. Participants reported fear of being stigmatized by others, which led to behaviors such as skipping medication or avoiding situations for worry about unintentional disclosure. All participants endorsed experiencing external stigma on the HIV stigma scale; however, only 3 of 10 participants reported experiencing enacted stigma when directly asked to describe an experience during in-depth interviews. Participants described how *SYV* helped them have “more confidence”, accept themselves, and incorporate positive coping skills such as relaxation (deep breathing) when they felt stressed. The findings suggest *SYV* helped YLWH accept themselves, develop positive coping methods, and identify and form peer social support; but stigma remains common. Descriptions of stigma were not recognized as such; experiences of enacted stigma were acknowledged by some participants. More research is needed to understand and measure mental distress and wellness as well as stigma in this population so that interventions may more accurately detect change in key outcomes.

## Introduction

In 2020, there were 3.4 million youth (ages 15–24) living with HIV (YLWH) globally [1]. The majority of adolescents (ages 10–19) living with HIV (89%) reside in sub-Saharan Africa; and over half live in five countries: South Africa, Nigeria, Kenya, Mozambique, and Tanzania [2]. Despite improvements in antiretroviral therapy (ART) and free access to HIV testing and treatment, the rate of decline in AIDS related deaths has lagged behind in this population, and AIDS-related deaths remain one of the leading causes of death of youth in Tanzania [1]. The causes of AIDS related deaths among YLWH include delay in HIV testing, disengagement from care, and poor adherence to ART; all three factors are associated with poor psychosocial wellbeing and HIV-related stigma [3].

Stigma is a detrimental social phenomenon where individuals with certain, discrediting attribute(s) are devalued and rejected by other members of society [4]. The HIV Stigma Framework proposes that people living with HIV (PLWH) experience and respond to HIV-related stigma through three main mechanisms: enacted, internalized, and anticipated stigma. Enacted HIV stigma is represented by past experiences of discrimination and prejudice such as isolation due to someone’s HIV status. Internalized stigma occurs when a PLWH accepts negative attitudes and perceptions towards those living with HIV and applies that negativity towards themselves. Anticipated stigma includes PLWH’s expectations of future discrimination and prejudice based on their HIV status. These stigma mechanisms can affect PLWH’s physical, psychological, and behavioral outcomes [5]. HIV-related stigma has been found to hinder ART adherence and can lead to psychological distress among YLWH [6, 7].

YLWH’s experiences of living with a stigmatized, chronic condition are compounded with the physical trademarks of the disease such as flat warts on the skin, stunting, or lipodystrophy. Lack of social support due to the death of one or more parent can make the experience even more overwhelming [8, 9]. YLWH have reported isolation, abuse, and poor familial connections that affect their mental health [10–12]. HIV-related stigma has also been linked to poorer quality of life among people living with HIV [13].

Given the documented relationship between HIV stigma and the mental health of YLWH, it is crucial to investigate further how HIV-related stigma manifests in YLWH in Tanzania. The aim of this study was to understand better the change in mental health symptoms and experiences with stigma among YLWH who completed the pilot *Sauti ya Vijana* intervention (*SYV*), an individually randomized group treatment pilot trial developed with YLWH to address their identified mental health and psychosocial needs [14].

## Methods

### Overview

This study was conducted as part of the original *Sauti Ya Vijana* (SYV) pilot intervention in the Kilimanjaro region of Tanzania. Quantitative data were collected between May 2015 and July 2019 through interview-assisted structured questionnaires with enrolled YLWH and demonstrated trends towards improved mental health, adherence and a 10% increase in viral suppression among the intervention arm compared to the control arm [15]. Qualitative data were collected from August through September 2019 through in-depth interviews with a subset of 10 YLWH who completed the intervention. The study was approved by Duke University Health System Institutional Review Board, the Kilimanjaro Christian Medical Center (KCMC) Research Ethics Committee, and the Tanzania National Institute for Medical Research.

### Setting

The study took place at two hospitals in Moshi, Tanzania: Kilimanjaro Christian Medical Center and Mawenzi Regional Hospital Care and Treatment Center. Both hospitals host a monthly Saturday clinic, “Teen Club,” for young people, age 11–24 years old, where they receive HIV medical care and support from their peers.

### Participants

Eligibility criteria for participating in the SYV intervention has been described previously [15]. In short, YLWH were purposively sampled and were eligible to participate in in-depth interviews (IDIs) if they were at least 18 years of age, had attended at least eight of the ten SYV group sessions, had completed an 18-month follow-up study visit at the time of interviews. In an effort to get a variety of experiences with depression, youth were also required to demonstrate a change of 4 points in either direction on their PHQ-9 scores from baseline to 18 months. However, due to recruitment challenges, this criterion was later amended to include participants who demonstrated a half a standard deviation change (± 2 points). Participants were contacted by research assistants via telephone to determine their interest in completing in-depth interviews. Participants who were interested and eligible provided written informed consent.

### Procedures

The SYV intervention has been previously described [14, 15]. In summary, the intervention was delivered in a group setting across 10 group sessions by trained young adult group leaders. Session topics included identifying and coping with stressful events, sharing difficult memories with others, identifying and coping with internal and external stigma, disclosure, and identifying support persons in their lives [14, 15].

### Measures

Quantitative measures included in this study were collected at the enrollment/baseline and 18-months study visits (approximately one year after completing the SYV intervention). The Patient Health Questionnaire (PHQ-9) was used to measure depression symptoms. This is a standardized depression screening tool that has been used and validated in several sub-Saharan African countries [16, 17]. The PHQ-9 is a nine-item instrument; items are summed, with total scores ranging from 0–27. Internal and External Stigma were measured using a modified 10-question, 4 item, Berger HIV stigma scale [18]. The total stigma scores ranged from 10–40, four internal stigma questions (range 4–16) and six external stigma questions (range 6–24).

In-depth interviews were conducted in Kiswahili using a semi-structured interview guide. The interview guide was developed using the protocolized manual and session themes of the SYV intervention, clinical expertise of the study team, and data from formative research [14]. Topics included participants’ history of mental health challenges such as depression (defined as losing interest in things they usually enjoyed or feeling hopeless), current challenges of living with HIV, their experience with stigma, and their overall impressions of the intervention during and after the SYV intervention. Interviews were conducted by a trained female qualitative researcher (FN) in a private room at KCMC, lasted approximately 60 minutes, and were audio-recorded. To reduce bias, the qualitative researcher was external to the SYV study team and was not present for the intervention and did not administer the quantitative questionnaires during the follow-up visits. Participants received compensation to cover the cost of transportation to and from the interview as well as a meal voucher (∼$2 equivalent).

### Analysis

Two trained research assistants fluent in Swahili and English listened to the recordings and translated them to English transcripts. Analysis followed a three-step process. First, a member of the study team (LM) listened to the audio recordings and wrote narrative memos summarizing each participant’s interview. After transcription, the same study member reviewed the English transcripts and edited the memos as needed to ensure they were comprehensive. Second, interview transcripts were uploaded to a qualitative analysis software, NVivo 12, and coded using an integrated inductive and deductive thematic analysis approach [19]. Transcripts were coded using structural codes developed from the topics of the interview guide. Then, each structural code was reviewed independently and coded for emergent themes. A second person coded two transcripts independently, and the transcripts were uploaded to NVivo to assess consistency in coding. After discussion and agreement on common themes, a final codebook was developed and applied to the remaining transcripts. Queries were conducted in NVivo to look at cross tabulations of themes across codes. After querying, analytic memos were written to summarize themes and enriched with quotes from participants.

Descriptive statistics, such as mean, standard deviation, and range, were used to summarize responses to the PHQ-9 and Berger HIV stigma scale at baseline and 18-months. Quantitative analysis was conducted using Stata 16 software (Stata Corp, College Station, TX).

### Inclusivity in global research

Additional information regarding the ethical, cultural, and scientific considerations specific to inclusivity in global research is included in the Supporting Information (S1 checklist).

## Results

Twenty-three youth, of whom 11 had a PHQ-9 score increase of ≥2 and 12 had a PHQ-9 score decrease of ≤2, were identified and contacted for in-depth interviews. Due to migration and inability to establish phone communication, only 10 YLWH were interviewed including four with a PHQ-9 score increase of ≥2 and six with a PHQ-9 score decrease of ≥2. This group represented 17% of the 58 youth randomized to receive the SYV pilot intervention and 20% of the 49 youth who completed their 18-month follow-up study visit. The average age of participants who completed IDIs was 21.6 years old with an age range of 18–25 years. Seven participants were male, and three participants were enrolled from the Mawenzi site. Mean (Standard Deviation) PHQ-9 scores were 7.3 (3.5) at baseline and 5.6 (5.0) at 18 months.

### Experiences with stigma

Mean (SD) total stigma scores were 23.3 (3.4) at baseline and 24 (4.8) at 18 months. With the exception of three YLWH, external stigma scores of participants who completed IDIs stayed the same or increased from baseline to 18 months. Mean external stigma scores increased from 14.8 (3.3) at baseline to 16 (4.5) at 18 months. The mean (SD) internal stigma scores were essentially unchanged at 18 months, 8 (1.8) in comparison to baseline, 8.5 (1.6).

During in-depth interviews, YLWH were asked to describe a time they experienced stigma. All participants endorsed experiencing anticipated stigma or believing other people will stigmatize them if they found out about their HIV status. Three participants described instances of enacted stigma where they were isolated within their homes, ridiculed in public settings within their community, or discriminated against by teachers and classmates in school settings.

*“The first challenge was stigma, which really tortured me in that time. They segregated me from the others and the family to the point of separating my plates, cups, and sleeping arrangements.”*–Female, 18 years old

When asked directly, seven participants denied ever experiencing stigma; however, they all would describe instances of stigma during other parts of the interview without labeling them as such. These unprompted descriptions of stigma were often tied to discussions of history of depression, ART adherence, or opportunistic infections (Table 1).

**Table 1:** Selected quotes highlighting participants’ descriptions of their experiences with stigma.

	Acknowledged experiencing stigma	Description of not experiencing stigma	Described an experience they reported as stigma	Described an experience, but did not name it as a stigmatizing event
Male, 24 years old	Yes		“I was stigmatized by the neighbors when I was young. I did not know if this stigma to me was because of my condition [HIV].”	
Male, 18 years old	No	“Personally, I haven’t [experienced stigma].”		“I was embarrassed when people would start seeing me on the road and say I had the virus [HIV].”
Male, 20 years old	No	“I have never thought on something like that [stigma]”		“I feel if I don’t take the drugs properly, I will be sick…because you can be sick with a cough that doesn’t end…. Rashes may appear [on your skin]. A person might ask you, ‘why are you just coughing? Why have the rashes appeared? What is the problem?’. Questions like that discourage me, and you feel ‘aah! has he discovered my status?’”
Male, 23 years old	No	“Personally, I have never been through stigma. I am afraid of it, that is why I avoid it. When I suspect there is a sign that [stigma] will be [present], I escape.”		“The challenge is people receiving my health status. That is a big challenge. Even the people that I work with are not well educated on AIDS. So, when you mention [HIV] virus to them, they see it as a very dangerous thing.”
Female, 18 years old	No	“Personally, I have never being stigmatized by a person because I have never disclosed to someone about my real condition or my health condition. Also, I have never stigmatized myself because at home people care about me. They love me.”		
Male, 25 years old	No			“I was not tested and told I have this problem [HIV]. Rashes used to appear on my ears. In school, my schoolmates secluded me, and I sat alone. That problem made me lose happiness”
Male, 19 years old	No	“I was told not to [disclose my status] to anyone. I should not share with friends because later on they will come to seclude me. The community will start to seclude me.”		
Female, 22 years old	Yes		“I was in form four at school before I discovered my HIV condition. I became so weak and wasted to the point where my fellow students were saying that I had HIV. They started stigmatizing me. They started stigmatizing me and segregating me to the point that they stopped reading with me or even eating with me. They refused to sleep in the same room with me’”	
Female, 18 years old	Yes		When I started high school, my aunt used to separate all the dishes I used…I did hard work in the house and sometimes they didn’t give me food.”	
Male, 21 years old	No	“I don’t trust any of my friends. I can’t trust them completely. Even if we help each other on anything, I can’t tell them about my condition. I have not thought about telling anyone yet.”		

**Fig 1:**
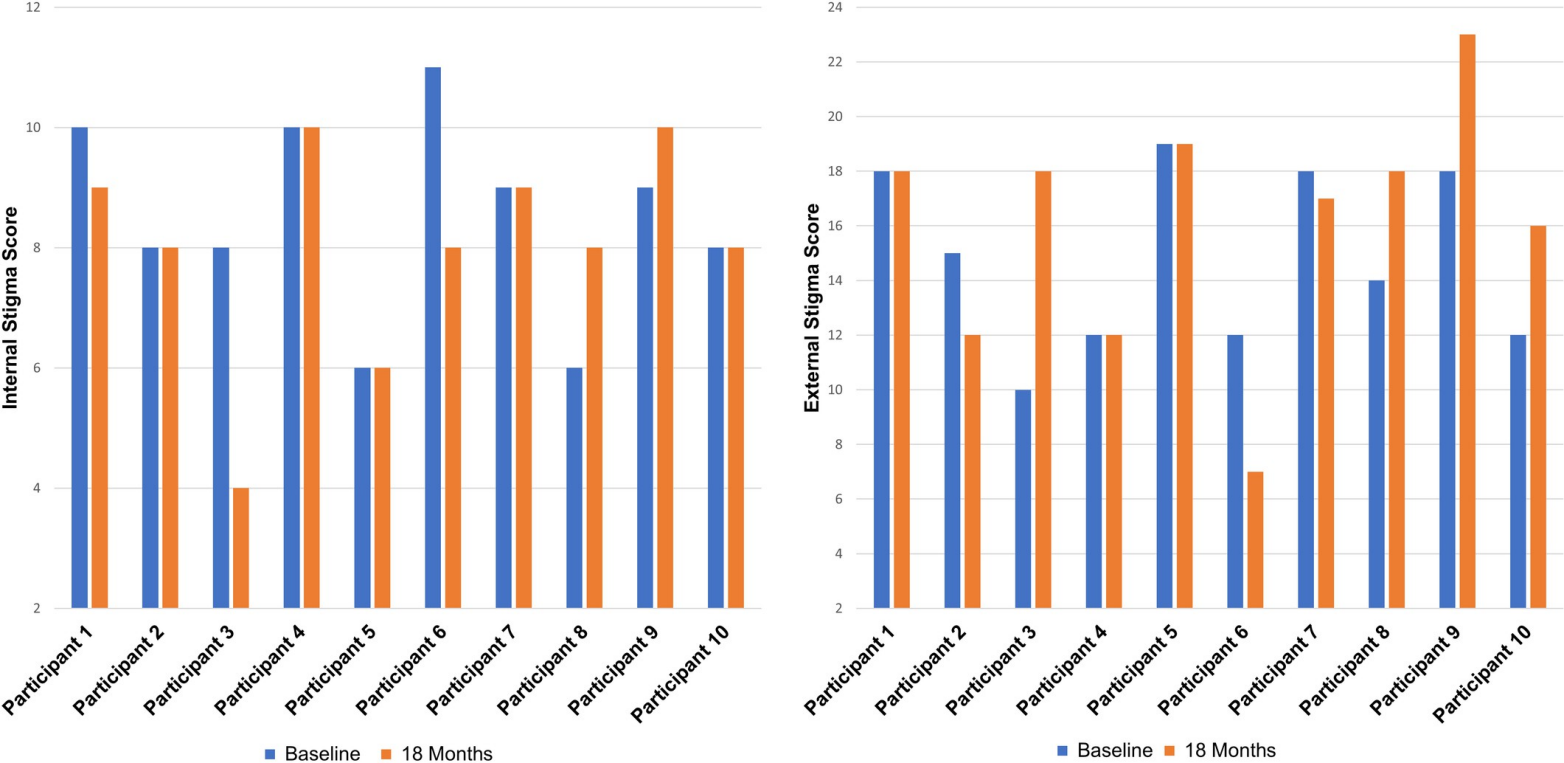
Modified Berger HIV stigma scale internal and external stigma scores at baseline and 18 months. Internal stigma scores range from 4–16 and external stigma scores range from 6–24.

Participants’ denial of experiencing stigma during IDIs contradicted their quantitative stigma scores as all participants endorsed experiencing some form of internal or external stigma on the Berger HIV Stigma Scale at the baseline and 18-month study visits (Fig 1).

### Mental health challenges

#### Triggers

During in-depth interviews, all participants reported experiencing intermittent episodes of mental health challenges, such as depressive symptoms, extreme sadness, or feelings of hopelessness, in the past. Only one participant endorsed feeling depressed at the time of the interview. Notably, YLWH described the ways in which stigma impacted their mental health; participants cited fear of unintentional disclosure and the resulting stigmatization as a prominent trigger of mental health distress. As a result, YLWH expressed losing hope about forming and maintaining romantic relationships and having children in the future.

*“On depression, there is one matter that is troubling me. I have a girlfriend that I love, but she is not HIV positive like me. So, how to tell her is difficult. It gives me thoughts and stress of what will happen if she [finds out]”–*Male, 23 years old

YLWH also expressed concerns of intentionally disclosing their status to siblings, family members, and friends. These concerns manifested in anxieties around taking ART. YLWH were uncomfortable with taking their medications in front of others and described how they concealed their medication use to prevent disclosure.

*“I normally prepare my drugs early. For example, if I have them in my pockets, I normally go outside without anyone knowing and take them. Or, if my drugs are in my bag, I will take my bag and leave. When I get outside, I will take the drugs and put them in my mouth. When I return inside, I will just take a cup of water and drink the water like normal. I will have swallowed my drugs without them knowing.–*Male, 24 years old

Relatedly, YLWH reported being worried about missing their ART dosing time, and some also reported occasionally skipping their medication when they were unable to take their medications in private. ART adherence concerns were also tied to fear of developing opportunistic infections and apprehension about developing “rashes”, tuberculosis, or a persistent cough, and wasting away as these are seen as markers of HIV infection in their communities.

Five participants identified difficult interpersonal relationships in their home life as triggers of mental health distress. These difficult relationships manifested in abuse, isolation, ridicule, and recurring arguments with other household members that were often centered around the participant’s HIV status.

*“…When I was living with my brother, sometimes, he gossiped about my condition when he was drunk…That made me lose peace because I wondered ‘why I am this way [living with HIV], and why is he doing that?’”*–Male 25 years old

Four participants reported experiencing suicidal ideation in the past, but these feelings were not present at the time of the interview. Thoughts of self-harm were reported to be brought on by interpersonal conflict or rejection by family members or romantic partners as a result of their HIV status.

*“I thought that I would be better off dead. On my father’s side of the family, my uncles didn’t want me. I wondered ‘why I should be alive and living in so much suffering and sadness?’”*–Female, 22 years old

#### Coping mechanisms

Participants had different methods of coping with stress and depression. Coping methods included listening to music, walking away from the stressful or triggering situation, and using skills they learned through the *SYV* intervention.

*"At the Sauti ya Vijana project we were taught to tighten like this (showing the interviewer) to reduce depression. So, I tried to use that, and it helped me reduce depression."–*Male, 21 years old

Peer social support, especially with other YLWH, was a coping mechanism endorsed by all participants. Participants with difficult relationships at home indicated that peer support helps them escape their problems at home and access psychosocial support that they would not receive otherwise. Additionally, for youth who were able to disclose their status to their friends, peer support also helped them cope with worries about medication adherence; and some participants reported using coded text messages to remind themselves and their peers to take their medication on time.

*“There are three friends of mine that are up here. We normally communicate and remind each other on the drugs. So, when the time comes, he reminds me and says, ‘my friend it’s time to take drugs.’.. I know completely a certain person will send me a message or call at certain time, so I carry my medication with me so that I’m ready.”*–Male, 20 years od

### Experience with SYV

All participants reported having a positive experience with the *SYV* intervention. They described “feeling free” during the intervention’s lessons. Participants also reported that *SYV* helped them accept themselves and their diagnosis and realize they were not alone. The intervention gave participants more courage to correct misinformation they hear in the community about HIV that could perpetuate stigma.

*“First, Sauti Ya Vijana has changed my life because it has made me accept myself. Second, I can’t hear a person talking badly about the issue of a person who has a condition like mine and leave him [alone]. I will have to talk about it in one way or another without him knowing I have [HIV].”* Male, 24 years old

Resilience, the ability to name and cope with difficult situations, emerged as one of the main long-term effects of SYV. In addition to incorporating coping skills they learned in SYV, participants reported that their ability to deal with challenges increased after participating in SYV.

*“For example, the challenge of stigma or maltreatment, Sauti ya Vijana has taught me how to avoid despairing and also how to be patient [with others].”*–Female, 18 years old

Specifically, participants were able to identify and choose healthier coping mechanisms, such as calming techniques and breathing exercises, for dealing with life challenges and mental health distress.

*“In the past, I believed that when you have a lot of thoughts [depression], drinking alcohol helps. But I did not know that when you drink alcohol it just helps for a short time, and you also get health effects. Now, I have completely stopped drinking alcohol.”–*Male, 23 years old.

Participants also reported using the skills they learned in SYV to help other people in their lives deal with mental health difficulties. One participant described sharing the lessons she learned in SYV with friends who did not partake in the intervention.

*“I know how to advise my friend against stigmatizing herself … Because of Sauti Ya Vijana, I normally deal with my challenges and even my relatives’ and friends’ challenges.”–*Female, 18 years old

## Discussion

YLWH navigate multiple challenges in their daily lives as a result of living with a stigmatized chronic condition. This study highlighted the ways in which YLWH who participated in SYV experience and cope with mental health challenges, perceive stigma, and incorporate the lessons from the intervention in their lives. YLWH in this study described challenges of living with HIV, such as fear of disclosure and stigma, while navigating difficult interpersonal relationships and mental health difficulties. Disclosure emerged as a theme that functioned dually as a source of distress and a means for YLWH to create peer support. YLWH avoided intentional and unintentional disclosure as a way to protect themselves from being stigmatized. Conversely, disclosing their status to other YLWH helped them develop a support system that served as a mechanism to cope with feelings of depression and facilitate ART adherence. These results are consistent with results from other studies that have demonstrated the dual effect of disclosure and importance of community and peer support in the mental health outcomes of YLWH [10, 11, 20–22].

Resilience emerged as an outcome among the youth who received the SYV intervention. Youth reported accepting themselves and their HIV status and increased ability to cope with challenges and mental health difficulties after the intervention. While resilience was not quantitatively measured in this cohort and in the larger trial, self-reported accounts from participants’ interviews demonstrate the potential that an intervention like SYV has in building resilience in YLWH [23]. Studies in other countries and in populations of older PLWH have had similar findings in improving resilience [24–26]. Additional research is needed to build evidence on effective methods of improving and measuring resilience in this population.

Our findings suggest that the way in which questions on the stigma scale are asked may not fully reveal the complexities of how YLWH experience or realize stigma. Though participants endorsed instances of internal and external stigma while completing the modified Berger HIV Stigma Scale, they did not readily recognize and name these experiences in the in-depth interviews. As reported previously, participants also didn’t recognize instances of internal stigma during the intervention’s stigma sessions [15]. Though the stigma scale used in this study has been used in many SSA countries, it is still based upon Western constructs. It is possible that stigma may have different meaning and interpretation in this cultural context, and the scale did not measure stigma in the study population as intended by the study team. As illustrated by HIV Stigma Framework and the responses in the in-depth interviews, anticipated stigma plays a large role in how YLWH perceive themselves and how they perceive stigma from the community [5, 27]. Yet, to date there are not many instruments that measure anticipated stigma. One promising tool is the Chronic Illness Anticipated Stigma Scale (CIASS); however, to the best of our knowledge, the scale has not been validated in YLWH in SSA [27]. Taken together, these findings indicate additional research is needed to understand further how YLWH in Tanzania experience, perceive and conceptualize the different types of HIV stigma to and identify the optimal and culturally contextualized methods of measuring stigma in this setting.

The results from this study also emphasize the need for community-targeted interventions to reduce stigmatizing attitudes towards people living with HIV. While the YLWH in this study cohort found ways to avoid and cope with HIV-related stigma and its resulting mental health distress, the systemic nature of HIV stigmatizing attitudes and discrimination must be addressed. Seclusion and prejudice in homes, schools, and other community settings highlight the dearth of safe spaces for YLWH [28]. Additionally, as demonstrated in other studies, stigma hinders ART adherence. In order to meet the goal of 95% of people knowing their status, receiving ART, and achieving viral suppression, addressing stigma as a barrier for ART adherence is crucial. There have been several interventions aimed at addressing HIV-related stigma within communities, health care workers, and PLWH [29, 30]. Adolescent specific interventions in Ethiopia, Nigeria, and Tanzania have been most effective in improving attitudes towards PLWH when schools have been used as intervention sites [31–33]. As stated previously, validated and culturally contextualized HIV stigma measures are necessary in order to develop and rigorously evaluate these stigma interventions.

These results should be interpreted in light of limitations. First, the study relied on self-report for all the variables. Because the quantitative and qualitative data were collected at KCMC, one of the recruitment sites where participants received their HIV care, there was a possibility of desirability bias. Second, the quantitative and qualitative measures were collected at different timepoints. We did not administer the PHQ-9 or the adapted 10-question Berger HIV Stigma Scale at the time of the in-depth interviews. As a result, the reported symptoms of depression and perceptions of stigma in the qualitative interviews were not contemporaneously evaluated with their quantitative scores. Third, due to challenges with establishing communication with SYV participants, the original eligibility criteria for interviews had to be modified in order to have an adequate sample size. The final sample did not represent the gender makeup and site distribution of the overall intervention group. However, despite these imbalances and the small sample size, no new codes emerged by the 10^th^ interview suggesting that saturation was achieved.

## Conclusion

The qualitative findings suggest *SYV* helped YLWH accept themselves, develop positive coping methods, and identify and form peer social support, but stigma remains common for this group. Descriptions of stigma were not always recognized as such, and only experiences of enacted stigma were acknowledged by participants. More research is needed to understand and measure mental distress and wellness as well as stigma in this population so that interventions can more accurately detect change in key outcomes.

## Supporting information

S1 Checklist PLOS questionnaire of global inclusivity. (DOCX)

S2 Checklist COREQ checklist. (PDF)

S1 Table Codebook. (DOCX)
