# Supplementary material for: Mental health challenges and perceptions of stigma among youth living with HIV in Tanzania
Source: PLoS One. 2025 Jan 28;20(1):e0318035. doi: 10.1371/journal.pone.0318035 (PMC11774362; doi:10.1371/journal.pone.0318035)
Supplement: S1 Table — (DOCX) [file pone.0318035.s003.docx]

| Code Name | Description | Supporting Quotes |
| --- | --- | --- |
| 1. Depression | Apply to any discussion of depression, extreme sadness, feelings of hopelessness, or suicidal ideation | “It was that of the death of my mother. I had depression and later on I encouraged myself. I just had to keep quiet and not share with someone.” -Male, 19 years old |
| a. History of suicidal ideation | Apply to participant responses on past or current experiences of suicidal ideation. Code will only be used for those endorsing or denying suicidal ideation | “When my father came home, he started scolding me. That is when I left, and it led me wanting to kill myself. I remember I took seven pills that day.” – Male, 21 years old  “...when I found out that my partner had another person. I felt very bad because I did not expect that... I honestly felt like the world was longer good. I felt between dying and living, it is better to die. God helped me.” -Male, 23 years old |
| b. Triggers | Apply to discussion of triggers or precipitating events of depressive thoughts, extreme sadness, feeling of hopelessness, or suicidal ideation | “I used to be depressed wondering, ‘oh my God! why did you take my mother away instead of killing me so that my mother would remain?’ This thing used to hurt me a lot in my soul” – Female, 22 years old |
| c. Coping | Apply to description of how youth cope with depressive thoughts, suicidal ideation, and life challenges | “ If I get depression, I don’t get upset to the point of stopping my medication. I just sit down and calm down until the depression goes away. Or, I go to my parents and explain my problem or another close person and who knows my situation. I tell them my problem and they give me ideas on how to cope with my depression.” – Male, 18 years old |
| 2. Challenges | Apply to descriptions of challenges participant faces in their life including HIV-related challenges |  |
| a. Fear of Disclosure | Apply to any discussion of fear of disclosure as a HIV-related challenge. Text can be double coded as 1B. Triggers | “I cannot disclose it to everybody because some people are gossip. They are likely to go and announce it to other people. Therefore, I select the people that I can tell about my condition” – Male, 18 years old  “I am not worried. I would be worried if someone discovers that I have [HIV], but, if a person does not know, I am not worried” -Male, 19 years old  “There are so many other people that I haven’t told. Some of them cannot keep secrets; they are gossips… they can start talking publicly and saying that so and so has HIV” -Female, 18 years old |
| b. Lack of social support | Apply to any discussion of lack of support systems or interpersonal issues as a challenge. Text can be double coded as 1B. Triggers | “The other challenge was lack of friends at school. At school, many friends used to avoid me because they knew that I had HIV. I lost most of my friends.” -Female, 18 years old |
| c. Finances | Apply to any discussion of finances as a life challenge. Text can be double coded as 1B. Triggers | “I mean, right now I don’t have depression. At the moment, I am free, and I am not thinking a lot…I used to worry a lot about where I was going to get a job and what I was going to do with the medicine. How was I going to manage it? But now I am working perfectly and using the medicine on time” – Male, 18 years old  “There are a lot of challenges. Apart from school challenges of studying, there is the life challenge of not having money for rent. Even at school, I ask my mother about money for books, and she doesn’t have money” – Male, 20 years old |
| d. Treatment adherence and pill burden | Apply to any discussion of challenges related to adherence to ART and pill burden. Text can be double coded as 1B. Triggers | “Other challenges are the normal ones maybe just taking drugs and things like that. Sometimes you are out with friends or at home with friends, and taking medication is a problem” – Male, 24 years old |
| 3. Stigma | Apply to any description of past or current experiences with stigma. The code will also be used to code participants’ denial of experiencing stigma | “ When you spend time with someone every day, you can observe their attitude when they speak about people with certain conditions [HIV]. So, you see that, if one day they found out about me, they will speak about me in the same way.” - Male, 23 years old |
| 4. Experience with SYV | Apply to participant’s description of their experience with the SYV intervention | “I first remember my peers. Some of them were very funny…I also remember it was a very free area for a person to share their story like the way I am sharing here regarding my depression. You can share, and people encourage you. I discovered that this situation is not just happening to me.” – Male, 23 years old |
| a. Coping skills from SYV | Apply to mention of specific coping skills they learn or use after completing the SYV intervention. Text can be double coded as 1C. Coping | “The training that we have been given is that when you have depression (or many thoughts), sit on a chair, straighten your legs, lean back on the chair. Then, you massage yourself slowly or someone massage you, and stress goes away” -Male, 19 years old M |
| b. SYV Lessons | Apply to discussion of specific SYV lessons. Also includes is participant does not remember lessons | “What helped to me was that the one who was teaching me in Sauti ya Vijana was sister XXX. When it was time for the sixth lesson about depression, they told us to come with her parents in order to reveal your HIV status. I had come with my aunt, and I told her everything about myself in detail” - Female, 22 years old  “Sauti Ya Vijana educated and entertained us. They educated us on different trainings , such as [different] types of stigma, that you can stigmatize yourself or be stigmatized. [We learned] there is something called thoughts, feelings and behavior.” – Female, 18 years old |
| c. Resilience | Apply to description of resilience in youth after completing SYV (examples include self-confidence, ability to cope with stressors, forming healthy relationships) | “ It’s that situation that has made me accept myself. You know, when you are told that you have that problem [HIV], you give up. After [SYV], I saw that I am like other people. I accept myself as me…it does not make me lose any happiness” – Male, 25 years old  “To accept that I have HIV. I learned that it was a normal condition). Though I am afraid of stigma, I don’t think of it much. Even if a person stigmatizes me, I can cut them off without a problem.” – Male, 23 years old |
